# Promoting Self-Care in Caregivers of Older Adults Living With Chronic Illness: The iCare4Me Study

**DOI:** 10.1093/geroni/igab046.1451

**Published:** 2021-12-17

**Authors:** Lauren Massimo, Karen Hirschman, Harleah Buck

## Abstract

Informal caregivers provide a substantial amount of social support to older adults which can be stressful and lead to poor self-care. When stressed, caregivers of persons living with chronic illness are less vigilant and less motivated to engage in self-care behaviors that are important for maintaining their own physical and emotional health. Support interventions can encourage self-care by helping caregivers to focus on values, solve problems, and transform their goals into action. In this symposium, we will discuss the iCareMe study, a randomized controlled trial (RCT) (NCT03988621) that tests a virtual support intervention which utilizes health coaching to increase self-care behaviors in caregivers of older adults living with chronic illnesses, such as heart failure and dementia. The first session will discuss the translation of self-care theory to the basis for the “Virtual Caregiver Coach for You” (ViCCY) intervention and will provide an overview of the iCare4Me randomized control trial designed for caregivers of persons living with advanced heart failure. The second session will describe the adaptation of the iCare4Me RCT to caregivers of persons living with dementia. The third session will highlight findings from a qualitative descriptive study examining the characteristics of effective health coaching used in these two RCTs. Finally, the last session will share findings from a cross-sectional analysis examining moderators of self-care in heart failure caregivers. Together, these presentations will illustrate the unique and innovative approach that iCare4Me has taken to improve self-care in caregivers of older adults living with chronic illness.

